# A Fibrin-Thrombin Based *In Vitro* Perfusion System to Study Flow-Related Prosthetic Heart Valves Thrombosis

**DOI:** 10.1007/s10439-024-03480-6

**Published:** 2024-03-08

**Authors:** Yevgeniy Kreinin, Yahel Talmon, Moran Levi, Maria Khoury, Itay Or, Mahli Raad, Gil Bolotin, Josué Sznitman, Netanel Korin

**Affiliations:** 1 Department of Biomedical Engineering, Technion—IIT, 3200003 Haifa, Israel; 2 Department of Cardiac Surgery, Rambam Health Care Campus, 3109601 Haifa, Israel; 3 The Ruth Bruce Rappaport Faculty of Medicine, Technion—IIT, 3525433 Haifa, Israel

**Keywords:** Prosthetic heart valve, Thrombosis, Hemodynamics, Fibrin clot, Recirculating flow

## Abstract

Prosthetic heart valve (PHV) replacement has increased the survival rate and quality of life for heart valve-diseased patients. However, PHV thrombosis remains a critical problem associated with these procedures. To better understand the PHV flow-related thrombosis problem, appropriate experimental models need to be developed. In this study, we present an *in vitro* fibrin clot model that mimics clot accumulation in PHVs under relevant hydrodynamic conditions while allowing real-time imaging. We created 3D-printed mechanical aortic valve models that were inserted into a transparent glass aorta model and connected to a system that simulates human aortic flow pulse and pressures. Thrombin was gradually injected into a circulating fibrinogen solution to induce fibrin clot formation, and clot accumulation was quantified via image analysis. The results of valves positioned in a normal versus a tilted configuration showed that clot accumulation correlated with the local flow features and was mainly present in areas of low shear and high residence time, where recirculating flows are dominant, as supported by computational fluid dynamic simulations. Overall, our work suggests that the developed method may provide data on flow-related clot accumulation in PHVs and may contribute to exploring new approaches and valve designs to reduce valve thrombosis.

**Supplementary Information:**

The online version contains supplementary material available at 10.1007/s10439-024-03480-6.

## Introduction

Heart valve disease is a condition where one or more of the heart's valves do not work properly. Fortunately, heart valve replacement surgery introduced over half a century ago has increased the survival rate and quality of life of patients with severe valvular disease. The most common valve requiring replacement is the aortic valve [1] through the use of two common types of prosthetic heart valves (PHVs), namely mechanical heart valves (MHV) and biological heart valves (BHV). BHVs tend to degenerate over an average period of ten years [2], but their use has significantly increased as they can be deployed via a minimally invasive transcatheter procedure. On the other hand, MHVs can last throughout the life of a patient but require lifetime anti-coagulant medication to reduce thrombotic complications [3].

**Fig. 1:**
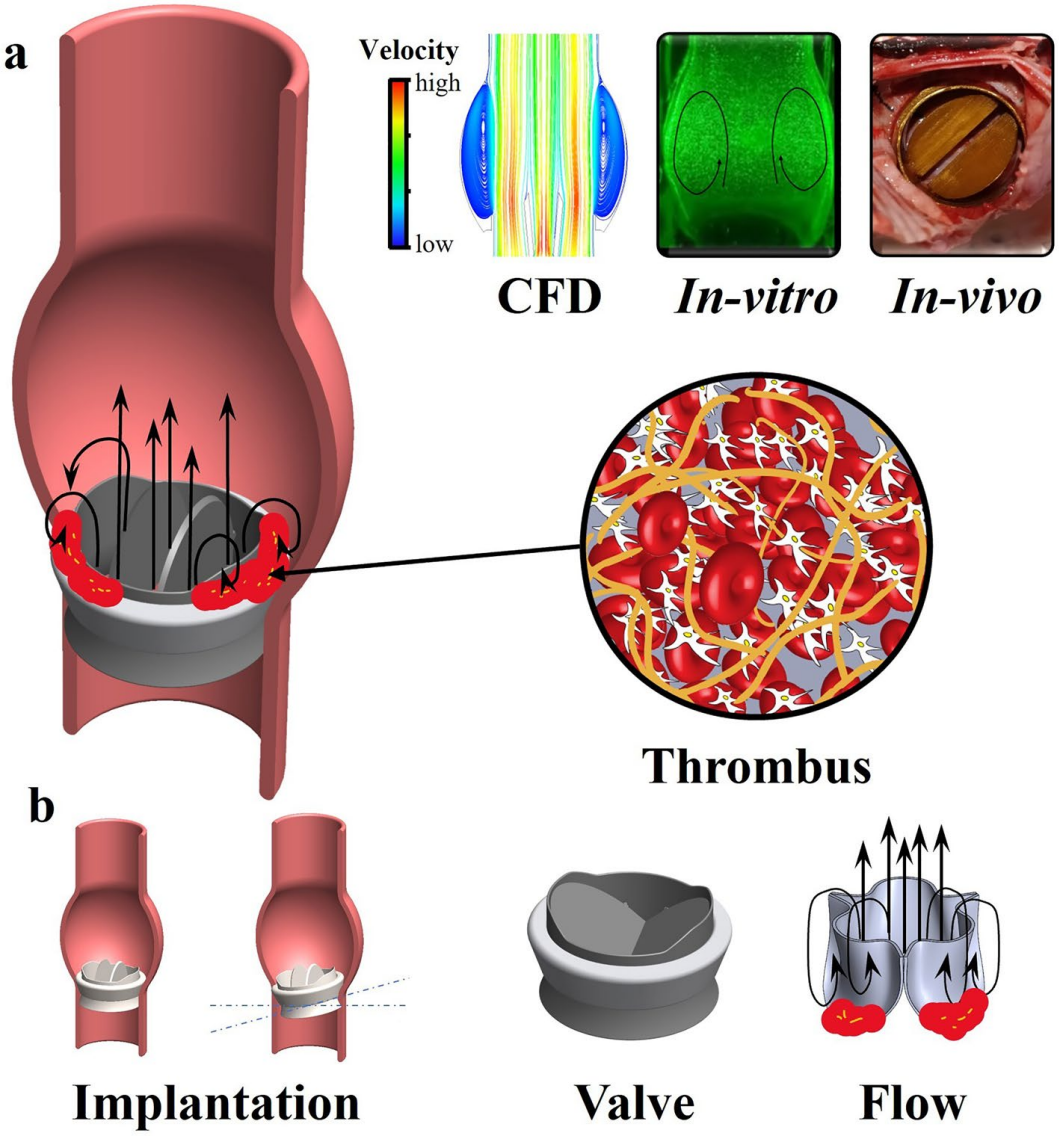
Flow-related heart valve thrombosis. **a** Left: Illustration of a mechanical aortic heart valve with a thrombus around the valve, flow around the valve includes recirculation areas near the sewing ring. Right: Heart valve thrombosis is studied by: CFD models, *In vitro* and *In vivo* experiments. **b** Heart valve thrombosis is affected by hemodynamic flow around the valve, the implementation configuration and the position of the valve, the patient’s blood composition and the blood flow profile

One of the main problems associated with aortic heart valve replacement, and MHVs in particular, is valve thrombosis [3]. Valve thrombosis is linked to abnormal hemodynamics in PHVs, resulting in high shear stress, which may lead to platelet activation and thrombosis initiation, and zones of low shear where clots tend to accumulate [4, 5]. Flow-related PHVs thrombosis is a multi-faceted biomechanical process governed by the complex local flow field around the valve (see Fig. 1a), which is affected by the specific PHV structure, its implantation configuration, the anatomic geometry around the valve, and the flow pulse, among other factors (see Fig. 1b).

To study and understand flow-related thrombosis in PHVs, computational fluid dynamics (CFD) simulations, *in vitro* experimental models, and *in vivo* studies are frequently sought (Fig. 1a). *In vivo* models are considered the ultimate test for PHVs function. However, large animal models are costly, complex, and limited in their ability to control the experimental conditions and perform flow measurements. Moreover, considerable variations exist in coagulation across different species, leading to instances where outcomes observed in animal thrombosis models may not accurately predict human thrombosis [6, 7]. Additionally, replicating valve thrombosis in animal models is highly challenging [8]. Conversely, CFD is probably the most widely applied method to explore PHV flow-associated thrombosis [9]. Using this approach, details of the complex flow features can be directly obtained and compared to *in vivo* and/or *in vitro* thrombosis experiments. For example, *in silico* studies have been applied to predict the risk and location of thrombus formation as a result of flow stagnation [10]. Additionally, CFD derived measures of residence time parameters have shown a correlation with *in vivo* thrombus patterns [11]. Other models focused on platelet shear activation and estimating a stress accumulation parameter of flowing platelets via integrating their stress loading [12]. In parallel to advances in PHV *in silico* flow modeling, *in vitro* experiments offer a method to experimentally validate and study flow-related thrombosis in a well-controlled environment, which can provide valuable insights into the mechanism of flow-related thrombosis. This latter approach is particularly useful for studying the role of various factors, such as wall shear stress and wall shear rate on thrombus accumulation [13]. Furthermore, *in vitro* models are attractive to validate results obtained from CFD simulations and *in vivo* studies and concurrently determine their limitations.

*In vitro* methods for studying PHV thrombosis include Particle Image Velocimetry (PIV) aimed to directly measure the flow field around the heart valve as well as functional flow assays aimed at mimicking the thrombosis event. Such assays can include the use of enzyme-activated milk, re-suspended platelet solutions, animal blood, or human blood. *In vitro* PIV studies have highlighted amongst others the unphysiological flows and prolonged flow stasis in the sinuses [14, 15] as well as allowed to explore the changes in flow using different procedures and devices [16]. In spite of the complexity in capturing local flow patterns in PHV in general, and in the neo-sinuses in particular, leading groups in the field have provided valuable insight into flows characterizing the neo-sinus via advanced PIV experiments [17–21]. Additionally, besides flow imaging experiments, functional flow assays can be informative in identifying thrombosis prone sites in PHVs. For example, Richardt *et al.* performed a PHV clot mimicking deposition study using enzyme-activated milk, which showed a clear similarity to the fibrin clotting effects on PHVs [22, 23]. However, this indirect approach, which is not directly based on a coagulation cascade, is complex to calibrate and perform [22, 24]. Experiments replicating PHV thrombosis using animal/human blood components can be highly valuable as they can functionally mimic the relevant processes in the coagulation cascade [25]. For example, due to the key role platelets play in PHV thrombosis, in which platelets are first activated by exposure to high shear and then get entrapped in low shear recirculation zones where they aggregate [26], several assays using resuspended platelets have developed to examine platelet shear activation [4, 27]. These methods have also included quantification of the procoagulant activity of platelets using a real-time assay of platelet activation state (PAS), based on a modification of the prothrombinase method [28]. Studies using the PAS assay in analyzing gel filtered platelet flowing in re-circulation loop based on a Left Ventricular Assist Device (LVAD) that perfused the platelets through a PHV, have provided valuable data on PHV thrombosis in a variety of valve configurations [29–32].

Other studies have utilized whole blood to examine clotting in PHVs. For example, Fallon *et al.* performed blood flow experiments study on various orifice geometries representing the MHV hinge region and studied how these geometries may contribute to platelet activation and thrombin generation [33]. Linde *et al.* developed a custom PHV thrombosis tester (THIA3) to perform experiments under physiological hemodynamic conditions using anti-coagulated porcine or human whole blood [34]. However, the use of relatively large volumes of fresh blood (>400 ml) remains technically challenging and can also produce variability between experiments [35]. Moreover, both blood and milk-based experiments do not allow direct imaging of the flow and processes that occur during the experiments.

Here, to overcome some of the limitations of current *in vitro* approaches and enable direct imaging of clot accumulation, we introduce an *in vitro* experimental approach based on human fibrin clot formation to mimic clot settling, and accumulation in PHVs—under relevant hydrodynamic conditions. In a first embodiment of the *in vitro* methodology, we explore a transparent, valve-containing aorta model to study fibrin clot accumulation on MHVs and monitor the valve function in real time. Specifically, we compare a normally implanted aortic MHV and a tilted implanted MHV and assess correlation between fibrin clot accumulation and local flow features, as extracted via CFD analysis. Although, the fibrin clot formation in the system is not directly induced by the valvular flow itself, our system allows to experimentally study, quantitatively and qualitatively, possible sites of clot settling and accumulation, governed by the mass transport characteristics associated with the PHV flow. Thus, in this context, our efforts can improve our understanding of PHVs’ flow-associated clot deposition and serve as an experimental tool for new implantation and device design improvements.

## Materials and Methods

### Materials

For the experimental system preparation, perfusion and cleaning the following materials were used: Dulbecco’s Phosphate Buffered Saline modified (D-PBS), without calcium chloride and magnesium chloride (Sigma Aldrich inc.) was used as the main solution in which fibrinogen was added; Human Fibrinogen (Enzyme Research Laboratories Inc.) was added to the PBS (45 μg/ml). Human a-Thrombin (Enzyme Research Laboratories Inc., 3,000 NIH unit/mg) was dissolved in PBS (30 μg/mL)and injected to convert fibrinogen to fibrin, see details in the Formation of Fibrin Clots Method section. For priming the system prior to clotting experiments; Bovine Serum Albumin (BSA) (Kankakee Inc.) was dissolved in PBS (1%) and perfused in the system. For washing and cleaning of the system following experiments; Sodium hypochlorite solution 120 g/L active chlorine (ADAMA Makhteshim inc.) was used. For the MHV and Aorta model manufacturing the following materials were used: ABS like resin (3DM Advanced Materials Inc.); Sylgard 184 22Kg kit (PDMS) (Dow Corning) ,Flat black paint (2X Ultra Cover Flat Spray, RUST-OLEUM Inc.), Transparent varnish spay (Jacobi Inc) as detailed in the Aorta Model and 3D printed MHVs method section.

### In Vitro Fibrin-Thrombin Perfusion System

**Fig. 2:**
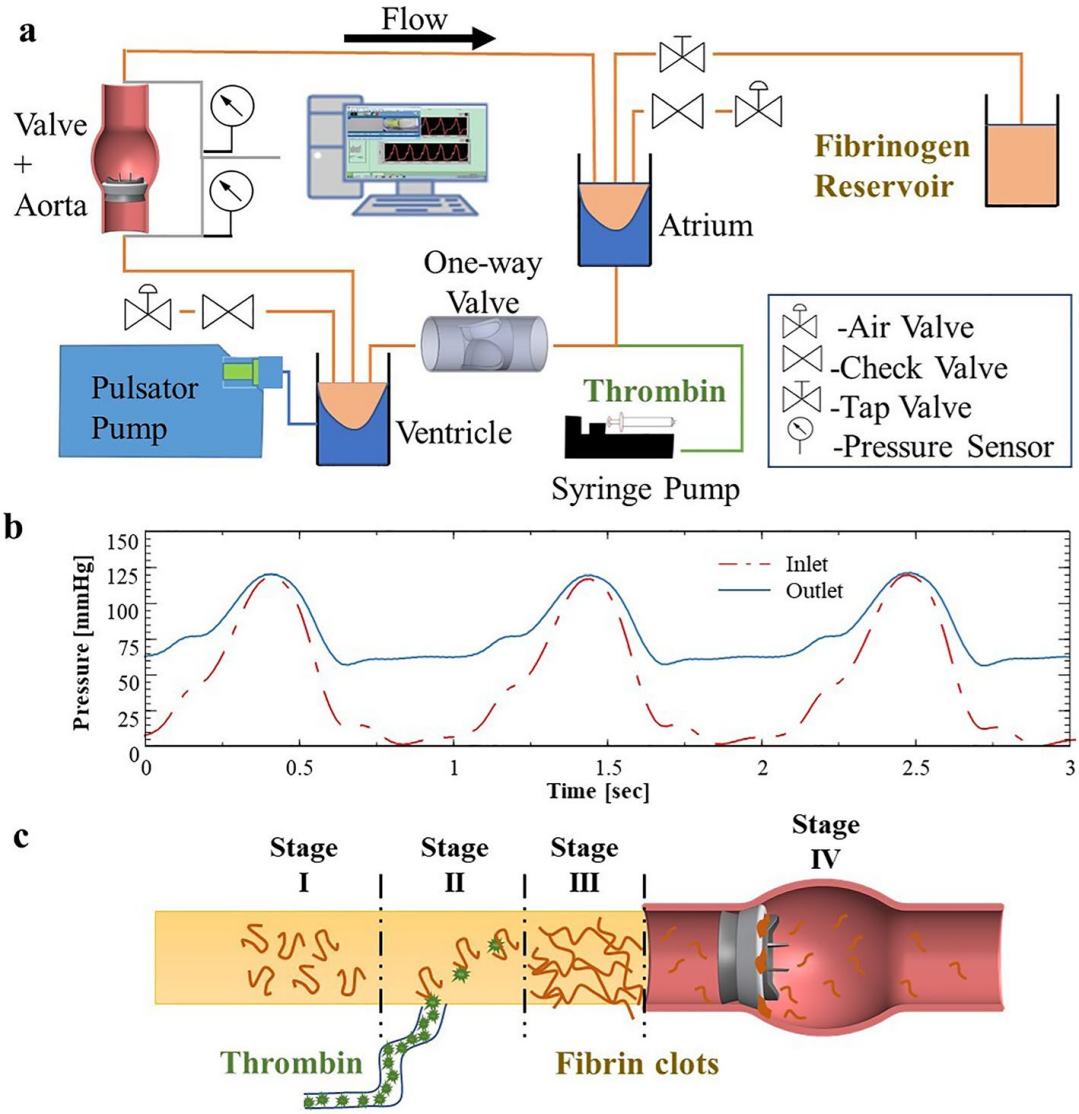
The fibrin-thrombin in vitro perfusion system to study heart valve thrombosis. **a** Schematic detailing the custom-built closed pulsatile experimental system mimicking physiological flow, enabling to monitor the pressures across the valve as well as to perform time-lapse imaging. **b** Pressure measurement at the inlet and outlet of an examined model in the system showing physiological pressure profiles. **c** Schematic of the fibrin-thrombin method applied in the experimental system, where a fibrinogen solution circulates in the system (stage 1), then injection of thrombin (stage 2) results in fibrin clot formation (stage 3) followed by fibrin clot settling in the valve (stage 4)

To emulate a realistic human heart aorta pulsatile flow, an experimental flow system was designed and built. Figure 2a shows a schematic of the experimental system, see also *Supplementary Materials* and photograph in Fig. S1. The system contains a pulsatile pump (Harvard Apparatus 665) modified by a 115cc hydraulic self-produced piston. The pump was connected to the simulated ventricle chamber, separated by a flexible sealed membrane made from a latex balloon (Jimitu Inc.), with one side of the tank connected via a tube to the pump and the other side is connected via a tube to the aorta model with the MHV. The inlet and outlet of the aorta model were connected to pressure sensors (PendoTech Inc., PREPS-N-000 PressureMAT Single-Use Sensor). The outlet of the aorta model was connected to the Atrium chamber, which comprises a flexible membrane (latex balloon) that is surrounded by another fluid (for details, see *Supplementary Materials*). The atrium was connected to the ventricle chamber through a 3D printed one-way valve. Air valves and directional valves were placed on the ventricle, and the atrium to prevent air bubbles in the system and drain it (see *Supplementary Materials* for details). Two web cameras (Blaupunkt BP-6310) were positioned to view the valve from above and from the side.

Under physiological flow, open-loop systems would require large amounts of fluids, and thus the system was designed as a closed-loop system similar to the circulatory system. To mimic the left ventricle and produce a controlled flow, a compliant latex balloon placed inside a fluid-filled chamber was externally compressed by its surrounding fluid via a pulsatile piston pump. The use of a compliant balloon allows dampening of the forces otherwise produced directly by piston pumps and can be valuable in reducing blood damage in future experiments with blood. Fluid injected from the ventricle chamber flows through a transparent aorta model, which includes the examined aortic valve implanted within it. The outlet of the aorta model is connected via a tube to a left atrium mimicking chamber, that consists of an elastic balloon, similar to the ventricle chamber. This balloon is connected via a tube to a reservoir to enable fluid filling during the pulse cycle. Fluid flows from the outlet of the atrium chamber through a one-way-valve to the ventricle chamber, thus replicating the flow between the left heart chambers which is regulated by the mitral valve. Altogether, this closed circuit allows to recapitulate the flow characteristics associated with the ventricle systole-diastole cycle. Additionally, it enables adjustable pressure gradients and physiological pressure curves via the use of a hydraulic pressure chamber that is connected to the surrounding fluid in both the ventricle and atrium chambers. Pressure sensors are located at the inlet and the outlets of the aortic model to monitor the operation of the valve. The examined valve is inserted into an aortic glass model, and real-time imaging is performed from two perpendicular views which allow to monitor clot accumulation on the valve during an experiment. The pressure sensors located upstream and downstream of the valve allow to monitor the pressure gradients throughout the experiments and ensure physiological pressure gradients [33].

The system flow conditions were defined to fit physiological values and set at 60 beats per minute (bpm), with a stroke volume of 100 ml, resulting in an average flow rate of 6 (L/min), performed at a 30/70 systolic to diastolic ratio. Measurement of the aortic pressure showed a 120-mmHg maximum and 60 mmHg minimum pressure pulse, while the ventricular pressure wave showed 120 mmHg maximum and 0 mmHg minimum values (Fig. 2b).

### Formation of Fibrin Clots

Prior to the clotting experiments, to passivate the surfaces in the system, the fluid reservoir was filled with 500 ml of 1% Bovine Serum Albumin (BSA) solution in D-PBS, which was perfused through the system for 1 h. A volume of 500 ml was used as it was sufficient to fill all the dead spaces in the system and allowed a stable physiological flow. Following this step, the BSA solution was emptied and in order to allow fibrin clot formation, the fluid reservoir was filled with a 500 ml human Fibrinogen solution (45 μg/ml in D-PBS). This Fibrinogen solution is the main fluid that circulates in the system during experiments, whereas upon interaction with locally infused thrombin, the fibrinogen transforms to fibrin, see Fig. 2c. The concentration of fibrinogen used in the system is much lower than its physiological level (20–40 mg/ml) but still allows formation of stable fibrin clots. Fibrin is a sticky protein that binds to other proteins and itself upon contact. To produce the fibrin clots, a thrombin solution (30 μg/mL, 90 U/ml), which approximately threefold higher than the threshold concentration (0.1 U/mL) required for fibrin formation [36], is slowly injected into the system at an injection port located just before the valve (flow rate 1 ml/hr using Harvard Apparatus Elite 11 syringe pump). This concentration of thrombin and injection flow rate resulted in timely stable formation of fibrin clots. This location of injection and the low flow rate ensure that the fibrin-forming reaction occurs in the region of the tested valve. As fibrin reaches the recirculation zones around the valve, clots form and accumulate around the valve.

### Aorta Model and 3D Printed MHVs

**Fig. 3:**
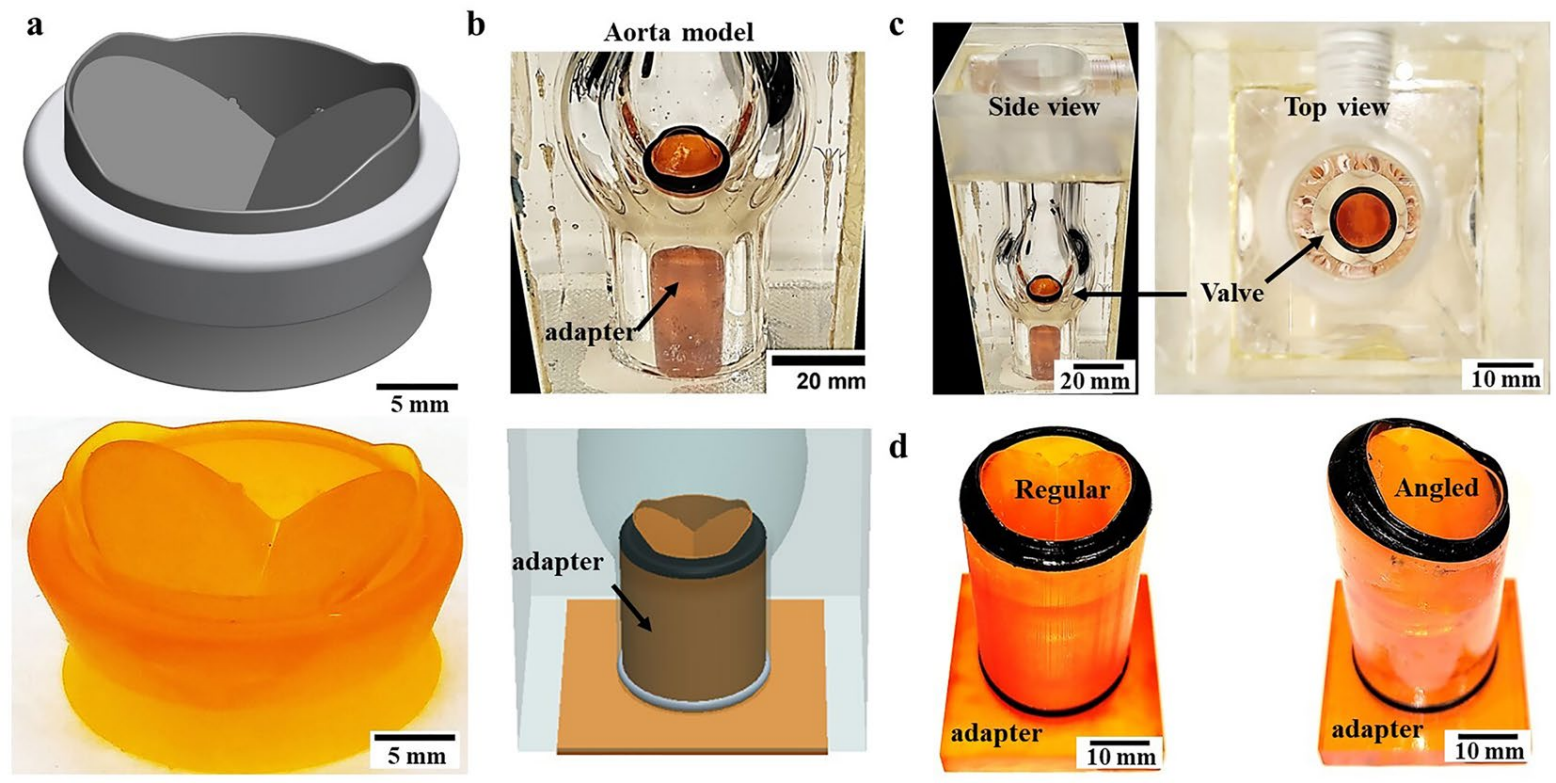
3D printed heart valve model in the transparent aorta model. **a** CAD model of a mechanical heart valve (top) and a photo of the 3D printed model. **b** CAD model and photo of heart valve model implanted inside the transparent aorta model (with black sewing ring) **c** Photo of a 3D printed valve in the transparent aorta model, left: front view, right: top view. **d** Two 3D printed configuration for valve implantation (Left- a regular positioned valve, Right- a tilted positioned valve)

The MHV model was designed using a SolidWorks® software based on published On-X manufacturer dimensions. The ONXAE−29 valve model with an external sewing ring diameter of 34mm was used (Fig. 3a). This size of the valve, which is the largest valve produced by the company, was chosen as it is less sensitive to the resolution limitations in 3D printed valves. Generally, the 3D printed valves require a low surface roughness and high printing resolution in order to reduce undesired clot accumulation due to rough surfaces and to produce functional valve hinges and leaflets. Accordingly, the valve was 3D-printed (Elegoo Mars) using a SLA technology 3D printer and ABS-like resin (Fig. 3b). The material used for printing, ABS-like resin (3DM), is brittle and exhibits no noticeable deformation due to the flow. As the model interacts with water, the resin also needs to exhibit low moisture absorption. The valve’s ring and the leaflets were 3D printed separately and then assembled. Following the 3D printing and postprocessing, the valve leaflets were heated to 100 °C, at which point the printed material becomes elastic, and the leaflets were inserted into the valve’s ring. After the assembly, the valves were covered with a layer of transparent varnish to reduce moisture absorption. To improve contrast of the fibrin clot and reduce their adhesion, the MHVs sewing ring was painted black and then covered with transparent varnish according to the manufacturer's instructions. The production process of the tilted valve was similar to the standard valve. To accurately position the valve within the aorta model, an anchoring element was designed and manufactured as part of the valve to ensure a fixed position for all valve configurations and experiments (see Fig. 3b). A guide tube was used to place the MHV at a fixed location in the orifice of the aorta, and an O-ring was used to provide sealing (Figure 3d). The aorta model was made of glass blowing a casted tube. The glass model was embedded in a rectangular box of Plexiglas and filled with PDMS at a 1:10 curing agent: base weight ratio (Fig 3b, c) allowing real-time imaging of the valve during its operation. In order to clean the MHVs after experiments, the MHVs were immersed for three hours in a solution containing 1% sodium hypochlorite, then washed with distilled water and left to dry overnight. In this first proof-of-concept, we explored both a normally upright implanted valve as well as a vertically tilted configuration, both using a similar adapter (Fig. 3d). For more details on the experiential setup, see the *Methods* section and *Supplementary Materials* (SM).For the MHV model used in this study, the aortic stroke volume was set to 100 ml/beat at a heart rate of 60 beats/min with a systole diastole ratio of 30/70 [34].

### Image Analysis

Image analysis was utilized to evaluate the clots accumulated on the valve. Initially, photographs of the valves were taken with time-lapse imaging every minute during the experiment. To produce a polar graph quantifying the amount of clots deposited as a function of angle, the valve image at the end of each experiment was used. For this analysis, the region of interest, specifically the sewing ring on the valve, was isolated using ImageJ® software. The grayscale threshold area of the clots was examined using the same software. Next, the pixel intensity of the valve in the grayscale area corresponding to the clots was evaluated and plotted by using Matlab® software. Furthermore, time-lapse images were used to observe the clots' accumulation as a function of time. The intensities were normalized, and results plotted. During the experiment, the intensities of the clots for each frame were evaluated, normalized, and plotted on an intensity graph as a function of time [37].

### CFD Method

We modeled the MHV in the aortic root using SolidWorks®. The models meshed in ANSYS GAMBIT 21 R1, and the simulations were conducted in ANSYS® fluent 21 R1 solving for mass and momentum conservation (i.e. Navier-Stokes equations). Blood flow was simulated under steady-state conditions and assuming a Newtonian incompressible fluid. The flow was assumed to be laminar based on the clinical data reported by Yamaguchi and Parker [38]. A fluid viscosity value of $3.5\times {10}^{-3} [kg/m\bullet s]$ and density of $1080 [Kg/m{m}^{3}]$ was used to simulate blood. The inlet tube was significantly extended to allow for the flow to fully develop prior to entering the valve and at the inflow boundary, a normal uniform velocity (1 m/sec) was specified as well as a constant pressure. Zero gradient conditions for the velocity components were set at the outlet boundary which was significantly extended to allow the outlet flow to recover. No-slip and no-flux conditions are specified for the velocity components on the rigid aorta walls and the PHV surfaces. For details on convergence test, see also Supplementary Materials and Figure S3.

### Statistical Analysis

T-test was performed to evaluate statistical significance, where *P* < 0.05 denotes statistical significance. All error bars are depicted as the Standard Deviation (SD). Prior to T-test analysis the Shapiro-Wilk test was employed to assess the normality of the data, and a significance level of *p* > 0.05 was chosen as the criteria to accept the hypothesis of normality.

## Results

### Spatio-Temporal Clot Accumulation Dynamics

**Fig. 4:**
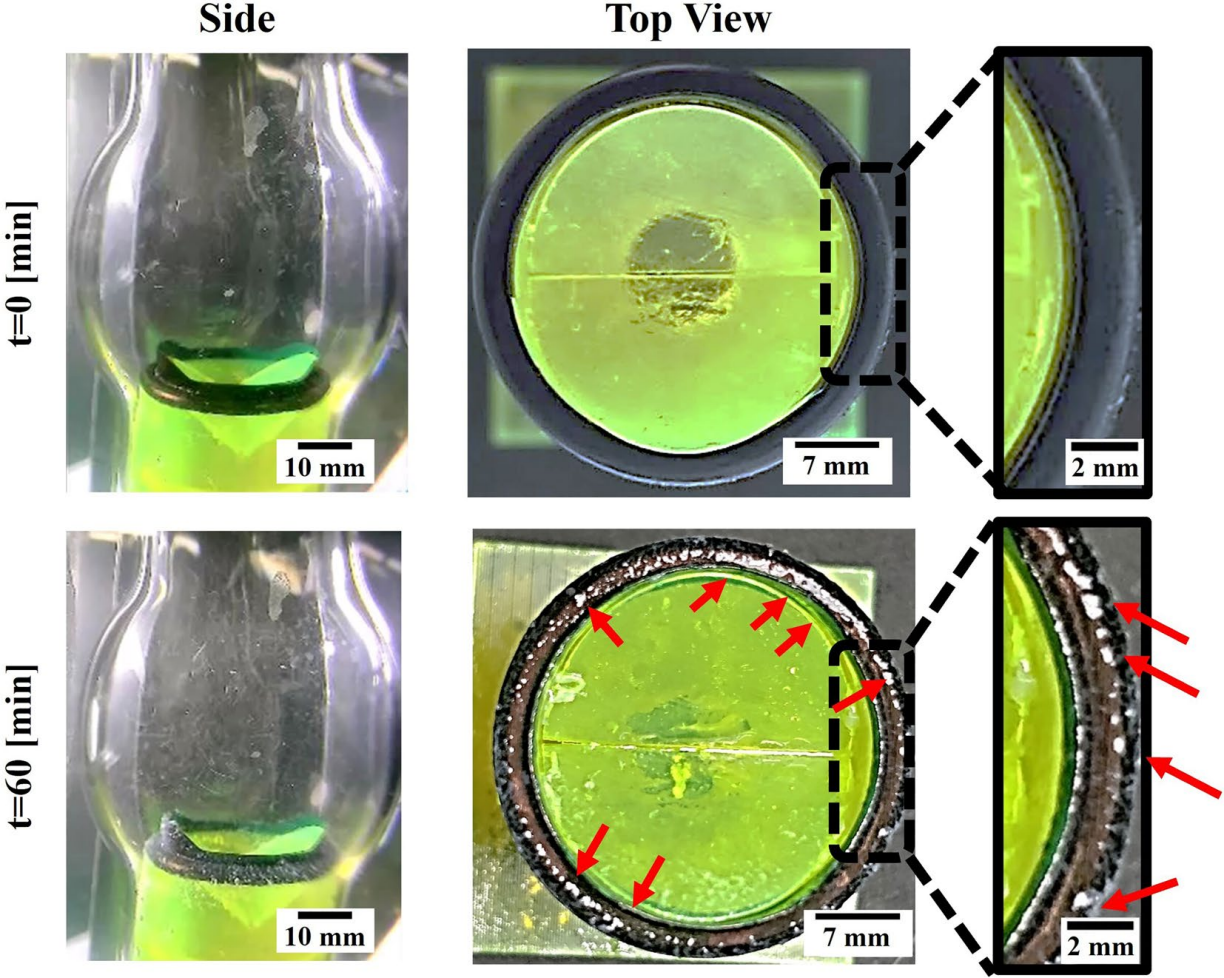
Spatial clot accumulation around a 3D printed heart valve model. Image of the 3D printed heart valve model inside a transparent aorta model prior to the perfusion in the system (*t*=0) and following 60 minutes of perfusion (*t*=60min) showing accumulation of clot at the sewing ring structure around the valve (see right insets). Red arrows point to fibrin clots

**Fig. 5:**
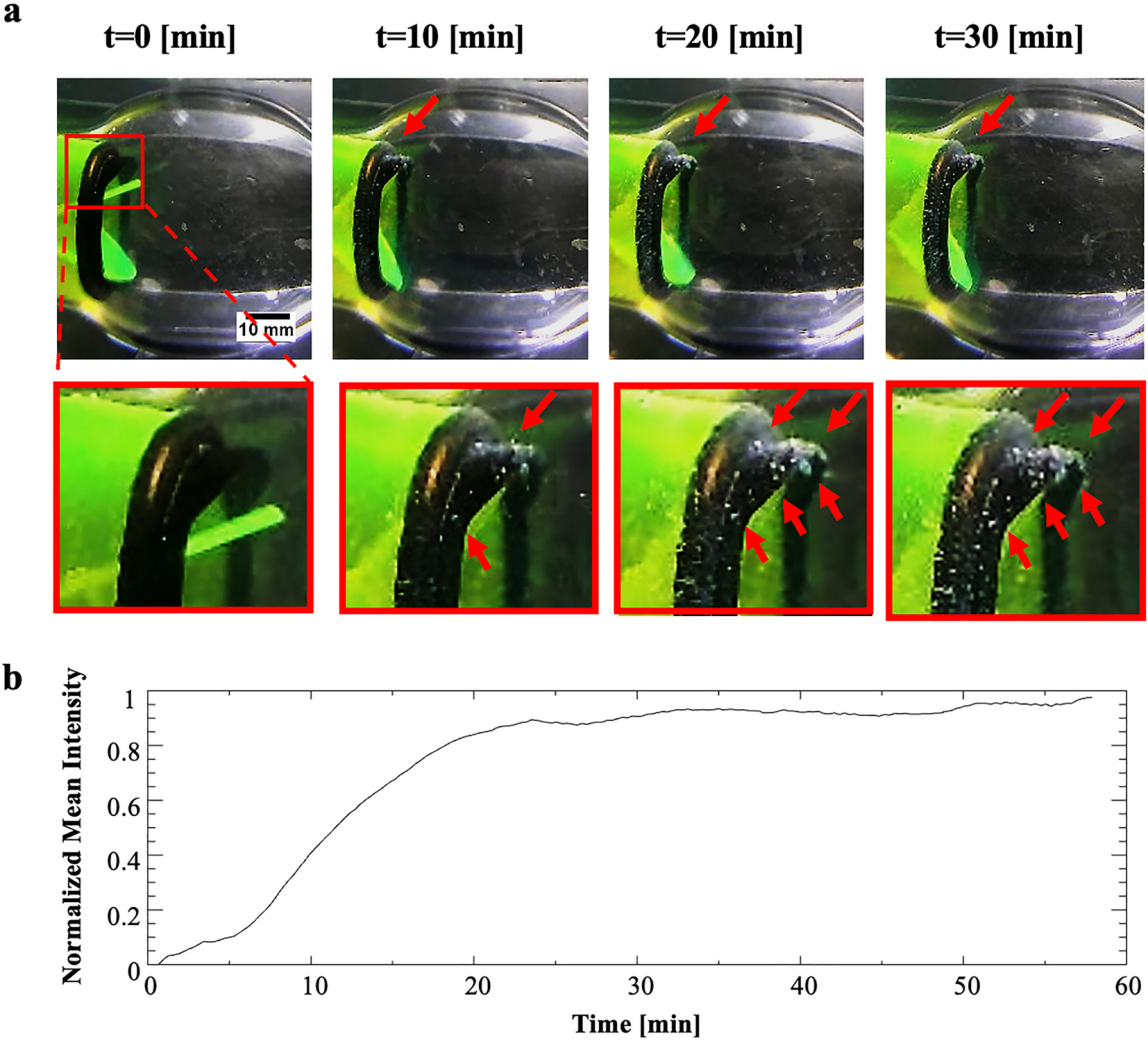
Temporal clot accumulation around a 3D printed heart valve model. **a** Side view time-lapse images of the 3D printed heart valve model inside a transparent aorta during a perfusion experiment showing the clot accumulation process over time. Bottom panel shows zoomed-in photos. Red lines point to fibrin clot accumulation spots. **b** Quantification of the normalized clot signal around the valve over time during the perfusion experiment showing an increased signal over 30 minutes followed by a plateau

The system allows studying spatially the accumulation of clots around a valve subjected to physiological flow. As shown in Fig. 4, clots show enhanced accumulation on the sewing ring around the MHV, see sideview (left) and top (right) image of the valve before and after 60 min perfusion experiments. Figure 5a shows time-lapse images during a perfusion experiment revealing the clot accumulation kinetics that increases over time, see also SM Movie 1. To quantify clot accumulation around the valves, front view images of the valves at the end of a 60 min perfusion experiment were collected and then threshold to detect clots, which exhibit a strong signal compared to the background signal. Following this step, the number of pixels corresponding to clots were quantified and normalized with the number of pixels representing the entire area of the valve. Figure 5b shows such quantitative analysis where clot accumulation saturates after 30 min of perfusion. Within 30 min under the examined conditions of clot concentration, clot size, and flow rates, we observed a plateau in the clot accumulation graph, we believe this is the result of a dynamic equilibrium between the amount of clot settling and clots being washed away by the flow. In addition to image analysis, weight measurements of the valve before and after the experiment were used to calculate the net added clots on top of the valve (see Fig. S2).

### Clot Accumulation Around A Normally Oriented MHV vs. A Tilted Valve

As a case study to examine clot accumulation under different conditions, two forms of implantation configurations were tested: a normally oriented valve and a valve that was vertically titled at 17° compared to its axisymmetric axis, see Fig. 3d. The value of 17° was chosen as it represents an arbitrary small angle (<20°) that can occur in practice but still has a significant effect on the flow field, which can affect the coagulation around the valve, as shown in previous studies on tilt angles between 10 and 20° [39–41].

**Fig. 6:**
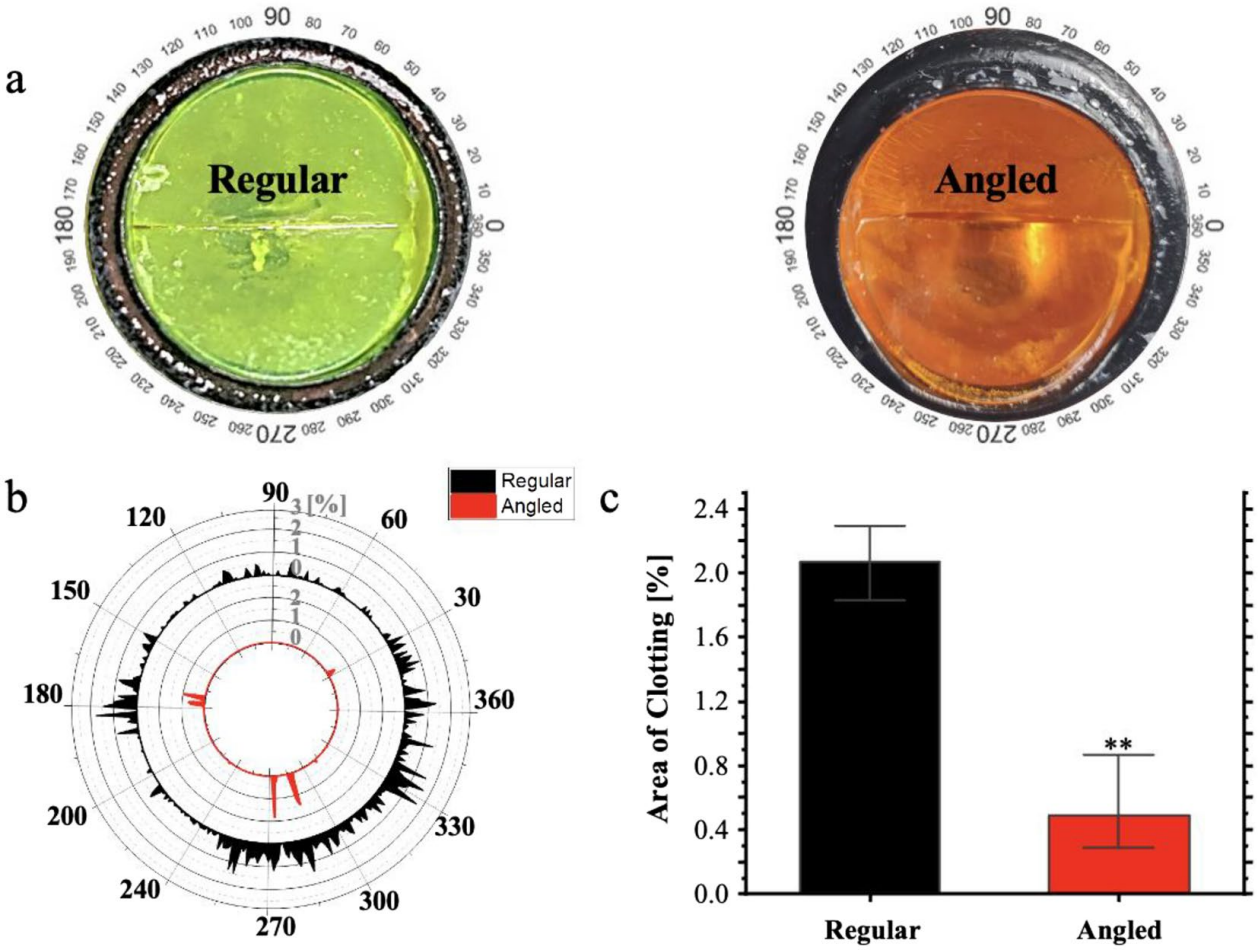
Clot accumulation around a regular vs. a 17° tilted valve. **a** Images of valve after 60 minutes of perfusion showing clot accumulation at the sewing ring of the valves - the regular orientated valve (top) to the 17° tilted valve (bottom). **b** An angular distribution graph of the presence of the clot covered area on regular vs. 17° tilted valve. **c** The overall percent of the clot covered area on regular vs. a tilted valves showing the significant decrease in clot accumulation around the tilted valve (*t*-test, *p*<0.05)

Representative images of the two valves at the end of a 60-minute perfusion experiment show that the tilted valve exhibits reduced clot accumulation at the sewing ring compared to the normal valve, see Fig. 6a. A graph presenting the angular distribution of the percent clot covered area (see angle notation in the figure) highlights that the regular valve (the outer circle) exhibits a higher area coverage of clots all around the valve compared to the angled valve (the inner circle). These results are consistent with the quantification of the clot covered area in both types of valves, showing a statistically significant difference between these two conditions, see Fig. 6c (*p* < 0.05, t-test). Additionally, these results are also supported by weight measurements of the valve before and after experiments where the tilted valve had a lower increase in the weight, suggesting a lower clot accumulation, see Fig. S2 in SM.

Moreover, for the normal implanted valve, the clot deposition areas around the valve qualitatively correlated with prior results shown in literature [35, 42]. As for the tilted valve, the result showed that the valve implantation orientation had a significant effect on the amount of clot deposition around the valve. Interestingly, the tilted valve showed a significant decrease in clot settling around the valve.

### Flow Analysis and Correlation with Clot Accumulation

**Fig. 7:**
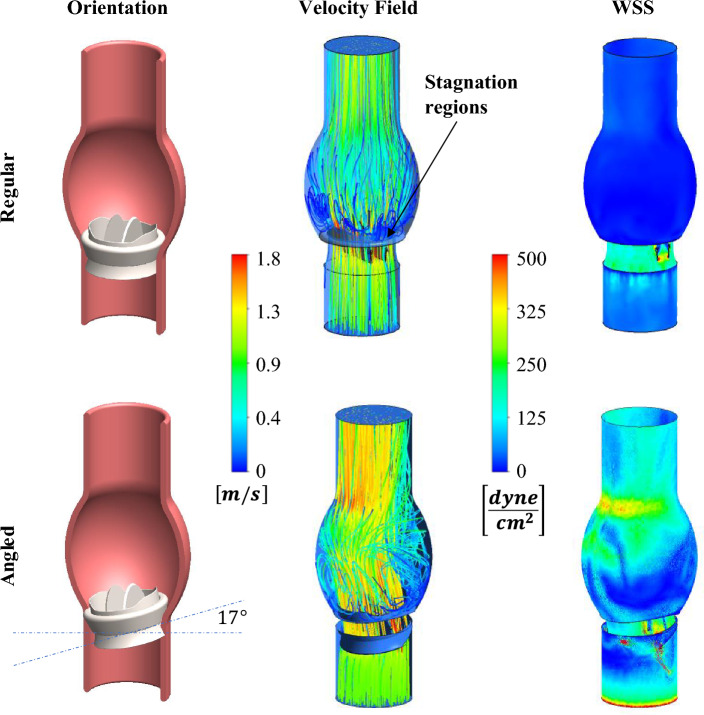
Computational fluid dynamic analysis of flow stasis in a regular vs. an angled oriented valve. Left: CAD image of the regular vs. tilted valve in the aorta (top–regular, bottom—tilted valve). Middle: 3D streamlines around the regular vs. tilted valve in the aorta model (top—regular valve areas, bottom—tilted valve) showing more stagnation zones around the regular valve. Right: The corresponding 3D CFD wall shear stress maps in the regular vs. tilted valve (top–regular, bottom—tilted valve) showing higher WSS around the tilted valve

The correlation between the clot accumulation results and the corresponding flow fields of the two tested configurations was investigated with CFD study. Comparison between Wall Shear Stress (WSS) maps on the aorta model in both conditions reveals larger sites featuring low levels of WSS (< 5 dyne/cm^2^) in the normal configuration, particularly around the sewing ring, whereas the level of the WSS at these sites in the tilted valve is notably higher (>80 dyne/cm^2^), see Fig. 7. The low WSS in the normal configuration suggests a low blood velocity around the valve and high particle residence time, as well as the existence of stagnation zones and, consequently, increased coagulation at these sites. In contrast, the increased WSS around the tilted valve, as per the conditions in our experiments, indicates higher local velocities that reduce clot deposition in this configuration. This is also supported by the streamline maps, as shown in Fig. 7, which shows low velocities and the existence of slow re-circulating flow structures in the normal configuration, whereas, in the tilted experimental configuration, these are less pronounced. However, it is worth to noting, that this finding is in contradiction to the general literature on tilted valves [39, 41, 43] , which shows that tilted valves are more problematic and prone the thrombosis. This is mainly due to the fact that our experiments do not account for platelets response which is important in PHV thrombosis; see discussion section on these results and the limitation sub-section.

## Discussion

### Fibrin-Thrombin Perfusion System and 3D Printed MHVs

The designed system, presented in this study, allows to produce the physiological conditions simulating the aortic flow with defined aortic stroke volume, pulse rate, and systole diastole ratio, and the valve can be positioned with different anchoring orientations inside the aortic root, thus enabling realistic testing of mechanical heart valve prostheses or bioprosthetic heart valves. Moreover, by using 3D printed valve models it is simple to examine different mechanical valve configurations in the system. The current experimental setup has several limitations, and improvements should be considered for future work -see limitation section for details.

In this study, in order to focus on demonstrating the developed method and to simplify the imaging and data analysis, we have used a simple aorta geometry as well as a nominal flow pulse. For example, the aortic model we utilized is an axisymmetric straight model and does not account for the complex geometry of the aorta, its asymmetric Valsalva sinuses and associated arterial branches. As shown in recent studies examining patient-specific aortic curvatures, the aortic curvature affects the flow in the sinus, where vortical flow patterns in the ascending aorta produce recirculation region that enhances the back flow into the sinus which can reduce flow stagnation [44]. Additionally, the three Valsalva sinuses of the aortic root, break the symmetry of the flow and affect the flow patterns the recirculation zones around the PHVs and thus directly influence clot accumulation [45]. In addition to these structural features that affect flow around the PHVs, the coronary artery flow valve [46] and aortic compliance [25], and can also affect PHV thrombosis.

Regarding the examined pressure/flow conditions, we focused on a nominal normal pressure pulse and heart rate, however, coexisting hypertension and aortic valve disease are frequent and a recent in vitro study investigated the effect changes in systolic and diastolic blood pressures on sinus hemodynamics showed that elevated blood pressure after BHV deployment affect the flow properties around the PHV [47]. Thus, it would be interesting to study in our system the effect of pathological pressure/flow on PHV clot accumulation. Additionally, the viscosity of the fibrinogen solution is 1.16 + /− 0.05 cP which is lower than blood viscosity and, in the future, Dextran can be added to solution to adjust the viscosity and serve as a blood analog with proper viscosity to match the pressure-flow relationship under blood flow.

### Spatio-Temporal Fibrin Clot Accumulation Dynamics

The results highlight that using the fibrin-thrombin model is possible to acquire quantitative spatial-temporal results evaluating the coagulation around the valve, while avoiding the complexity and inconsistent results associated with blood or whole milk experiments [35, 42, 48, 49]. Moreover, the system allows real-time imaging and pressure measurements to monitor both the operation of the valve as well as the clot accumulation during an experiment [50]. So far, in previous valve thrombosis studies, the quantification of the results was based on weighing the valve models after the experiment and by indicating the locations of the clots on the valves via visually comparing these locations to the areas of the clot accumulation on the valves of patients. In our model, quantification of the results is performed via image analysis allowing to have quantitative spatial results on the location/distribution of the clots. Moreover, usually *in vivo* quantification of thrombus deposition in valve prosthesis studies is done by autologous platelet radio-labeling which does not provide spatial information on the deposition [51]. On the other hand, here, it is possible to spatially map the deposition pattern, which can be valuable in improving the design of new PHVs.

### Flow Analysis and Correlation with Fibrin Clot Accumulation Around a Normally Oriented MHV vs. A Tilted Valve

The flow analysis of the low WSS regions, which are known to correlate with increased coagulation, correlated with the finding in our experimental model that the tilted valve, as per our experimental setup, exhibits less low WSS regions and lower clot accumulation. However, it is important to note that the tilted valve results in a jet directed toward the artery’s wall, as shown in the CFD analysis. Such as, jet produces high WSS (see region with WSS > 100 dyne/cm^2^) and flow impingement that is pathological and can be harmful as well as lead to platelet activation which will facilitate PHV thrombosis. These findings have also been reported previously when the flow field around a 20° tilted valve was numerically analyzed and compared to a standard valve, highlighting that this tilt angle can potentially result in platelet activation due to high shear stress [52], which will encourage valve thrombosis. The authors of this study concluded that the pivot axis of the valve should be implanted parallel to the aortic root’s curvature in order to minimize platelets activation. Other studies had similar conclusions on the effect of a tilted valve on platelets activation, which was suggested to support free emboli formation [41]. Hence, the inconstancy between the increased thrombosis risk associated with tilted MHV and our experimental findings suggest that a fibrin-thrombin assay, as per the existing experimental setup, is restricted to scenarios involving non-tilted valves. Thus, performing experiments using whole blood or using platelets suspended in plasma would be valuable in providing an accurate picture of the thrombotic and embolic risks associated with mis-oriented valves.

### Limitations

#### Lack of Platelets and Surface Contact Activation

As mentioned previously, platelets play a pivotal role in PHV thrombosis and analysis of circulating platelets through PHVs, as performed by leading labs in the field is important to properly study and understand PHV thrombosis. As the current perfusion model was designed utilizing compartments mimicking the heart operation and using indirect deflection of deformable and soft membranes, it can potentially also be used in the future to perfuse platelet without causing undesired side effects associated with mechanical blood damage, such as platelet shear activation.

Additionally, as platelets or recalcified blood are not perfused in the system, it does not account for surface contact activation and its potential effect on PHV thrombus formation [3, 53]. On one hand, this allows us to use 3D printed models that are composed of material which are not blood compatible and that can be easily produced and thus allow the study of different valve designs in a simple manner. On the other hand, contact activation and the biomaterial aspects of valve design are fundamental in development of PHVs and in PHV thrombosis. Thus, for both in vitro blood contact studies and in vivo studies, valves need to be produced with proper blood contacting materials and coatings, as is the case with pyrolytic carbon used in the On-X valve [54].

#### CFD Analysis

Experimental validation of the CFD results is valuable to re-assure the CFD results and, as the model is transparent and allows imaging, in the future Particle Image Velocimetry (PIV) studies can be performed to characterize the flow field. The PIV results could also be used to confirm the CFD model. Additionally, more complex CFD models that include prediction modules for coagulation formation and coagulation settling can be used [41]. Moreover, these models should incorporate studying turbulent shear stresses existing in PHVs that have been correlated with blood damage and platelet activation [55]. The CFD analysis in this study was done under steady-state conditions when the valve is fully opened, and the leaflets are in a fixed position. However, to accurately describe the flow field throughout the cardiac cycle, the simulation should include Fluid-Structure Interactions (FSI) that would allow to simulate the valve’s motion and the pulsatile flow field [56].

## Conclusions

In summary, the presented Fibrin-Thrombin based *in vitro* perfusion system may be valuable for evaluating the flow-related thrombotic potential of heart valve prostheses in the aortic position. It provides various options to evaluate valve performance and thrombosis under physiological, anatomic, and structural hemodynamics (e.g., hemodynamics and thrombosis of different valve structures and mis-orientated valve implantation configurations). In the current study, we focused on MHVs, but the system can also be utilized to study sub-clinical clot accumulation in BHVs [57–59]. More generally, the fibrin-thrombin model can be valuable in experimentally revealing sites that are prone to flow-associated clot accumulation, however, the model uses only two key components in thrombosis and lack other important players like circulating platelets. Thus, the system can be applied, in the future, to explore platelet activation and whole blood thrombosis, which can be highly valuable in studying PHVs flow-associated thrombosis.

### Supplementary Information

Below is the link to the electronic supplementary material.

Supplementary file1 (DOCX 1361 KB)

Supplementary file2 (MP4 53229 KB)

## Data Availability

All data needed to evaluate the conclusions in the paper are presented in the paper and/or the Supplementary Materials. Additional data related to this paper may be requested from the authors by contacting the corresponding author (N.K.).
