# Supplementary material for: A Fibrin-Thrombin Based In Vitro Perfusion System to Study Flow-Related Prosthetic Heart Valves Thrombosis
Source: Ann Biomed Eng. 2024 Mar 8;52(6):1665–77. doi: 10.1007/s10439-024-03480-6 (PMC11082030; doi:10.1007/s10439-024-03480-6)
Supplement: Supplementary file 1 — Supplementary file1 (DOCX 1361 KB) [file 10439_2024_3480_MOESM1_ESM.docx]

**A Fibrin-Thrombin based *In Vitro* Perfusion System to Study Flow-Related Prosthetic Heart Valves Thrombosis**

**Annals of Biomedical Engineering - Supplementary Material**

**Yevgeniy Kreinin**^1^**, Yahel Talmon**^1^**, Moran Levi**^1^**, Maria Khoury**^1^**, Itay Or**^2^**, Mahli Raad**^2^**, Gil Bolotin**^2,3^**, Josué Sznitman**^1^**, and Netanel Korin**^1^*

1 Technion - IIT, Department of Biomedical Engineering, Haifa, 3200003, Israel

2 Rambam Health Care Campus, Department of Cardiac Surgery, Haifa, 3109601, Israel

3 Technion - IIT, The Ruth Bruce Rappaport Faculty of Medicine, Haifa, 3525433, Israel

* E-mail address of the corresponding author: korin@bm.technion.ac.il

**Design of the perfusion system**

**
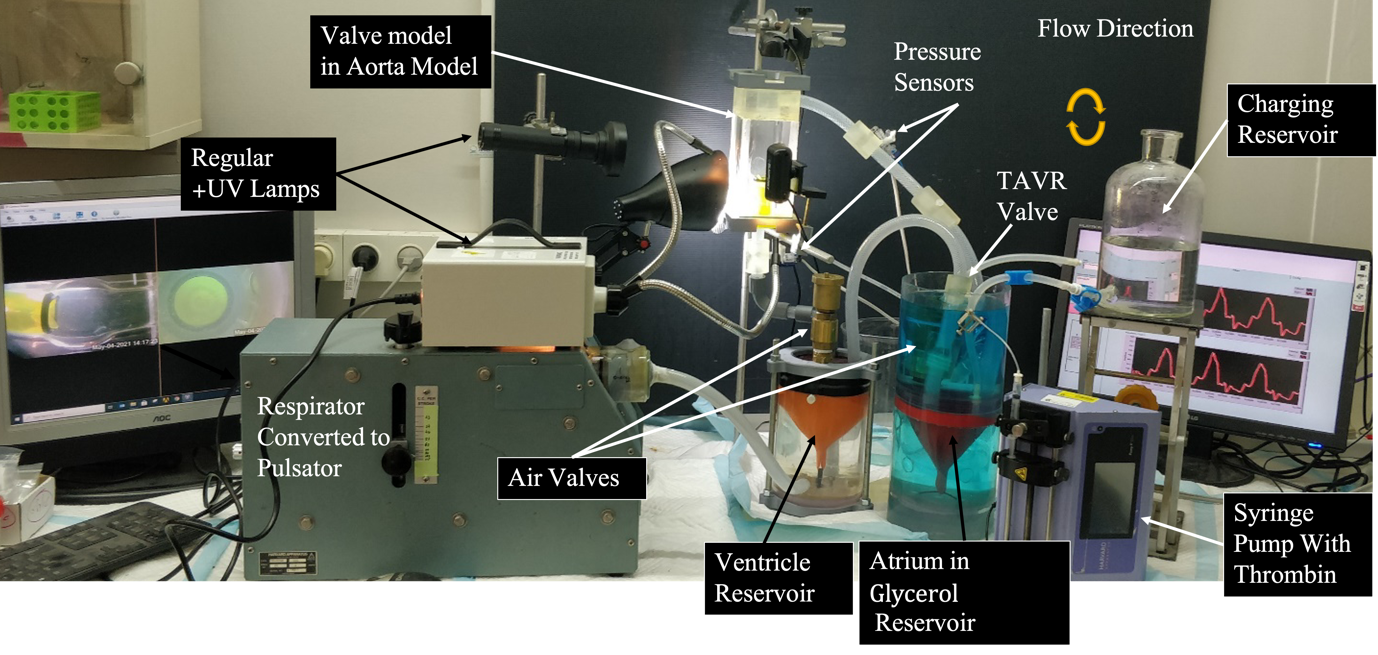
**

***Figure S1: The fibrin-thrombin in vitro perfusion system.***  *Detailed photo of the custom-built closed pulsatile experimental system mimicking physiological flow and allowing to monitor the pressures as well time-lapse imaging.*

Figure S1 shows a photo of the perfusion experimental system. The system includes a pulsator made from an animal ventilator (Harvard Apparatus Inc. Model ‘665’ Intermediate Ventilator MA1 55-0798). The pulsator is connected to the hydrostatic pressure ventricle reservoir and drives the system. The ventricle mimicking chamber is made of a balloon (orange in image) and connected to an air valve designed to remove air from the system, an inlet tube from the atrium mimicking tank, and an outlet to the tested valve model. Pressures are used to measure the pressure across the valve model. The tested valve is placed in an aorta model and visualized in real time, allowing coagulation and normal operation to be monitored. The monitoring of the valve is displayed on the second screen of the system (on the left). The aorta model is connected to the atrium reservoir. The atrium container is placed in a glycerol solution, which creates additional hydrostatic pressure on the atrium balloon (red) and helps it squeeze the atrium fluids back into the ventricle. The atrium has two inlets, one from the aorta model and the other from the flow reservoir which is intended to fill the flow fluids into the system. The atrium has two outlets, one is used to connect the air valve to discharge residual air from the system, and the other connects the atrium to the ventricle. On top of the connection between the atrium and the ventricle, a 3D printed one way valve is connected, which functions as a mitral valve. In addition, there is a connector designed to enable the injection of thrombin into the system prior to the entrance to the aortic chamber. A syringe pump controls the perfusion of thrombin through this connector [1].

**Clot accumulation weight measurements**


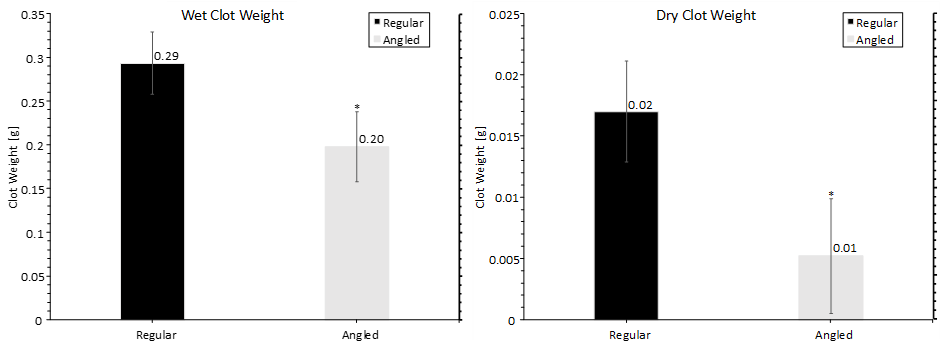


***Figure S2: Wet and dry clot weight results for a regular and 17 degree tilted valve.*** *Results show an increased clot mass in the regular valve. It is worth to note that the measurement shows high variability, although there is statistical significance between the groups in both measurements (p<0.05, t-test).*

The mass of the clot accumulating was evaluated via weight measurements. Weighing measurements can be done immediately after the experiment, when the clots are still saturated with liquid, or after drying the valves and clots. We performed the measurements by weighing the valve model before, immediately after, and after drying at room temperature for 24 hours. Weighing in a dry state ensures that drops/moisture do not remain trapped on the valve body and distort the results. However, as the change in weight for dry clots are in the order of micrograms, their accuracy is highly problematic; therefore, wet clots were also measured to reduce these deviations. The results of the weight measurements agree with the results of the imaging analysis, and Figure 6 shows a significant reduction in the clot accumulation around the tilted valve [2].

**CFD analysis and converges tests**

| **Geometry** | **Element Type** | **Number of Elements** |
| --- | --- | --- |
| Regular coarse mesh | Polyhedral | 21K |
| Regular medium mesh | Polyhedral | 35K |
| Regular fine mesh | Polyhedral | 37K |
| Angled coarse mesh | Tetrahedral | 36K |
| Angled medium mesh | Polyhedral | 7K |
| Angled fine mesh | Polyhedral | 6K |

***TABLE S1 -The examined mesh types and number of elements*.**

***Figure S3: Regular and Angled valve simulations convergence graphs****. The convergence graphs were made by sampling the pressure located on the valves model symmetry axis. The graphs show converged simulation results and mesh independence at the studied mesh.*

To compare the flow maps between the normal and the angled implanted valve, a study was carried out using CFD. For this purpose, a polyhedra mesh was made on both models and tested under constant flow conditions, including gravitation forces and assuming laminar flow. In order to test the convergence of the studies to a low error value and test the reliability of the results, each model was tested by generating three different mesh densities to show convergences of the results, see Table S1. These studies included tests of the convergence of continuity values, velocity, the arrival of the entry and exit values of the flow, and the convergence of the average vorticity value in the area of the valve sewing ring, to convergence residual criteria of${10}^{-6}$. Also, the comparison of the pressure along the symmetry axis of the model was examined, see Figure 3S.

**Supplementary Movie S1**

**Movie S1:** Time-lapse imaging movie showing the clot accumulation around a 3D printed heart valve over a 45-minute perfusion experiment (1 sec represent 1 minute in real-time).

**References**

[1] T. Linde, J. Clauser, B. Meuris, and U. Steinseifer, *Assessing the Thrombogenic Potential of Heart Valve Prostheses: An Approach for a Standardized In-Vitro Method*, Cardiovascular Engineering and Technology **10**, 216 (2019).

[2] S. H. McKellar, J. L. Thompson, and H. V. Schaff, *A Model of Heterotopic Aortic Valve Replacement for Studying Thromboembolism Prophylaxis in Mechanical Valve Prostheses*, Journal of Surgical Research **141**, 1 (2007).
